# Molecular Characteristics of High-Grade Glioma in Relation to 5-Aminolevulinic Acid (5-ALA) Fluorescence Intensity

**DOI:** 10.7759/cureus.77774

**Published:** 2025-01-21

**Authors:** Santiago Garfias-Arjona, Monica Lara-Almunia, Ester Antón-Valentí, Javier Pierola-Lopetegui, Joan Bestard-Escalas, Albert Maimó-Barceló, Diego M Marzese-Parrilli, Sandra Íñiguez-Muñoz, Miquel Ensenyat-Mendez, Marta Brell

**Affiliations:** 1 Neurosurgery, Hospital de Llevant, Manacor, Spain; 2 Neurosurgery, Hospital Quiron Son Veri, Llucmajor, ESP; 3 Neurological Surgery, Fundación Jiménez Díaz, Madrid, ESP; 4 Pathology, Son Espases University Hospital, Palma, ESP; 5 Research and Development, Health Research Institute of the Balearic Islands, Palma, ESP; 6 Research, Health Research Institute of the Balearic Islands, Palma, ESP; 7 Cancer Epigenetics Laboratory, Cancer Cell Biology Group, Health Research Institute of the Balearic Islands, Palma, ESP; 8 Neurosurgery, Son Espases University Hospital, Palma, ESP

**Keywords:** 5-ala, aminolevulinic acid, brain tumors cns tumors, fluorescence, glioblastoma, high grade glioma (hgg)

## Abstract

Introduction: 5-aminolevulinic acid (5-ALA) fluorescence used in glioma surgery has different intensities within tumors and among different patients, some molecular and external factors have been implicated, but there is no clear evidence analyzing the difference of fluorescence according to glioma molecular characteristics. This study aimed to compare molecular factors of glioma samples with fluorescence intensity to identify potential cofounders and associations with clinically relevant tumor features.

Methods: Tumor samples of high-grade glioma patients operated using 5-ALA for guided resection were included for comparative analysis of fluorescence intensity and molecular features. All the samples were processed under the same conditions. The power for fluorescent stimulation and acquisition time was the same between samples. An inverted fluorescence microscope compared the mean fluorescence for each molecular variation. p53, ATRX and Ki67 expression and IDH1 mutation were assessed by immunohistochemistry. Follow-up of the patients for progression-free survival and overall survival was made.

Results: We found that the fluorescence intensity for each specific tumor was independent of the methylation of the methylguanine-DNA-methyltransferase (MGMT) promoter region assessed by pyrosequencing, there was no association of fluorescence with p53, ATRX, IDH1 mutation as assessed by immunochemistry. Also, fluorescence intensity has no relation with time of tumor recurrence or overall survival.

Conclusion: With the results, we argue that many factors are involved in fluorescence intensity that may be related to the specific metabolic status of the glioma cells analyzed, which is more likely to be responsible for the variation of fluorescence.

## Introduction

High-grade glioma (HGG) is the most common malignant primary brain tumor. Despite the gold standard treatment, the overall survival (OS) is only 15 months. The most important factor associated with survival is the extent of resection. Molecular analysis has been used for prognosis. IDH1 and IDH2 mutations are associated with a better prognosis and are the most important biomarkers. Other biomarkers are associated with differences in prognosis. In IDH1 wildtype glioblastoma the presence of ATRX mutation is associated with favorable outcomes, while TP53 mutation is associated with worse prognostic. The presence of methylguanine-DNA-methyltransferase (MGMT) methylation confers a better prognosis as it indicates a better response to alkylating agents such as temozolomide. Other molecular markers, such as TERT mutation and estimated glomerular filtration rate (EGFR) amplification when presented together are associated with poorer prognosis. A better functional status at diagnosis is also correlated with a better prognosis.

5-aminolevulinic acid (5-ALA) is an endogenous substance that is orally administered and metabolized by glioma cells, among other cells. The metabolite protoporphyrin IX accumulates inside tumor cells and fluoresces when stimulated by blue light. Visible fluorescence can be used to identify the infiltrative borders of tumors and improve gross total resection. The fluorescence goes beyond the margins demarcated by contrast-enhanced magnetic resonance (MR) images.

The fluorescence intensity is extremely variable in tumor samples, as reported previously [1,2]. Fluorescence variability can affect tumor resection as there can be tumor areas with low or absent fluorescence that can be left after surgery. Fluorescence intensity has been associated with increased malignancy [2,3] and depends on the intrinsic [3-6] and environmental properties of the tumor [7,8].

Although fluorescence has been analyzed according to molecules involved in the home pathway, there is no evidence that one molecule alone is responsible for PPIX fluorescence. In addition to affecting home metabolism molecules, NADPH reduces fluorescence, as it reduces PPIX accumulation by activating home metabolism. In this study, we wanted to quantify the fluorescence intensity and its relationship with tumor properties and survival outcome to clarify if stronger fluorescence is related to a worse or better prognosis or a specific molecular tumor subtype.

The study was previously posted on a preprint form on Research Square in February 2024.

## Materials and methods

Forty-six patients with lesions suggestive of glioma in a reference center between 2014 and 2018 were enrolled. All the patients underwent open craniectomy for biopsy (n=9) or resection (n=37) using 5-ALA for tumoral visualization. HGG was confirmed upon histological examination, with three cases of grade 3 glioma and the remaining cases of grade 4 glioma.

After three to five hours of 5-ALA oral administration (20 mg/kg dose), a tumor sample from the most intense fluorescence region was obtained using a Pentero® surgical microscope with a blue 400TM module (Zeiss®, Oberkochen, Germany). The samples were frozen with liquid nitrogen and stored at -80°C until processing. The samples were cut using a microtome to obtain 10-µm thick slices with a Leyca CM3050S cryostat at -20°C, fixed with 1:1 acetone: methanol, and subsequently mounted in Dako fluorescence mounting medium.

To avoid differences between each sample analysis, they were processed following the same microscopical configuration for image acquisition. Samples were stimulated with blue light (wavelength 525-550 nm) using an inverted fluorescence microscope (D1-Cell Observer Zeiss®, Oberkochen, Germany). The power for fluorescent stimulation was set at maximum (HXP 120C) in the halogen lamp, acquisition time at 8965 ms, and emission fluorescence intensity was quantified in arbitrary units (a.u.) with the dsRED filter (605-670 nm) in 10 different tissue regions per sample using Zenn 2.3 Soft Blue software (Zeiss®, Oberkochen, Germany). Differences between registered intensities in the samples were assessed by statistical comparison of their mean and standard deviation. 

The tumor samples for histological analysis were processed with a standard protocol for formalin-fixed paraffin-embedded tissue. The occurrence of mitosis, necrosis, and microvascular proliferation was analyzed via hematoxylin-eosin staining for the diagnosis of Grade 3 or 4 HGG. Immunohistochemistry (IHC) analysis of p53, ATRX, and Ki67 expression and IDH1 R132H mutation status was retrospectively performed if available. Overexpression of p53 was confirmed if more than 10% of the tumor cells were positively stained, as indicated in the 2021 WHO classifications. The MGMT promoter methylation status was determined by pyrosequencing in some samples, and a cut-off value of 30% was used for determining hypermethylation status, as previously reported [9]. The Ki67 proliferative index was dichotomized as less than or more than 25% of cells positive for Ki67, which is associated with prognosis according to some studies [10,11].

Clinical data included age at diagnosis, progression date, date of death, or last follow-up (with at least 40 months of follow-up), type of surgical approach (biopsy vs complete resection), and type of treatment (Stupp regimen, radiotherapy, or chemotherapy alone). When proton MR spectroscopy was performed before surgery, the Cho/Cr ratio was calculated, and a threshold of 2 was used for analysis.

For statistical analysis, qualitative variables are described in terms of frequencies and percentages and compared using a nonparametric test. The mean value was determined for quantitative variables. OS was considered the time from the date of diagnosis to the date of death or the last follow-up. PFS was considered the time from the date of diagnosis to the date of clinical or radiological progression or death. The fluorescence intensity for each histological variable was compared using a nonparametric test for the mean difference between groups. For OS and PFS, samples were divided by mean values as follows: high fluorescence and low fluorescence.

K‒M curves for OS and PFS were generated for comparisons of clinical and histopathological characteristics using the log-rank test. A p-value of <0.05 was considered to indicate statistical significance. The statistical analysis was performed using IBM SPSS software, version 26 (IBM Corp., Armonk, NY).

For fluorescence intensity and its relation to clinical and histologic characteristics, the mean value was compared using a nonparametric test. For survival analysis, fluorescence intensity was dichotomized according to the mean value compared using the log-rank test.

For analysis of gene expression that could be associated with fluorescence, a PubMed/MEDLINE-based systematic review was performed by combining the main medical subject headings “5-ALA” and “fluorescence intensity.” A list of genes related to protoporphyrin metabolism and membrane transporters of metabolites was generated. Those whose expression had a clear positive or negative relationship with PPIX fluorescence were included in the analysis (SLC15A1, SLC15A2, CPOX, NOS1, NOS2, NOS3, GLS with a positive relationship and ABCB1, ABCG2, FECH, Frataxin, Mitoferrin-1, Mitoferrin-2, ALA-S2, ALA-S1, ABCB1, HIF1a, GLS2, MDH1, MDH2, SHMT2, MTHFD2, and MTHFD1L with a negative relation to fluorescence).

Using ATAC-seq HGG data from The Cancer Genome Atlas, a synthetic centroid was generated to assign a score to each patient. This classifier used the expression levels of the 23 genes related to the metabolism of 5-ALA. These genes were stratified as “positive” or “negative” depending on their effect on this process. “Positive” genes positively affect the uptake of 5-ALA by GBM cells, while “negative” genes have the opposite effect. A score was assigned for each gene according to the relative expression of the gene compared with that of the other genes. Patients with the highest number of “positive” genes had the highest score, while patients with the highest number of “negative” genes had the lowest score. The final score for each patient was computed by adding the score for each gene. The R/M3C package was used to create Uniform Manifold Approximation and Projection (UMAP) for dimension reduction representations using the gene expression levels of the 23 genes. The probability of fluorescence intensity was analyzed according to HGG molecular subtype, sex, and IDH mutation status. K‒M curves were plotted using Kaplan-Meier plotter.

Research ethics

This study was performed with the consent of the Ethics Committee of Investigation. Patient consent was obtained before surgery for sample analysis.

## Results

A total of 46 samples were collected. The mean age was 58.4 years at diagnosis (range 24 to 84 years), and 16 patients were older than 64 years. Radical surgery was performed on 37 patients. Forty-one patients received Stupp regimen treatment (60 Gy total radiation dose and 75 mg/m^2^ temozolomide), three patients did not receive any treatment because of rapid deterioration after surgery, one patient only received radiotherapy, and another patient only received chemotherapy because of her clinical situation after surgery (Table 1).

**Table 1:** Patients’ characteristics at diagnosis RT: radiotherapy, CT: chemotherapy, IDH-1: isocitrate dehydrogenase 1, MGMT: O6-methylguanine-DNA methyltransferase

Patients at baseline	n (46)	%
Sex		
Male	25	54.3
Female	21	45.7
Age		
<64 years	30	65.22
>64 years	16	34.78
Surgery		
Radical	37	80.4
Partial/biopsy	9	19.56
Treatment		
RT or none	4	8.6
RT-CT or CT alone	1	2.1
Stupp regimen	41	89.13
IDH-1		
Wild type	42	91.3
Mutated	4	8.7
MGMT methylation		
>29%	6	24
<29%	19	76

The median follow-up was 764 days (25.5 months), the median OS was 734.2 (24.5 months), and the median PFS was 428.1 days (13.0 months). For sex groups, there was no difference in PFS (449.7 days for females and 407.68 days for males, p=0.58) or OS (732.57 days for females and 735.24 days for males, p=0.51). PFS and OS differed according to age group (554.9 days PFS for the group under 64 years and 191.81 days for the group older than 64 years, p=0.003; OS of 889.9 days for the group under 64 years and 441.75 days for the group older than 64 years, p=0.019).

IDH-1 was mutated in four patients (8.7%), and the prognosis was better in the mutated group than in the wild-type group, although the difference was not statistically significant (PFS median time of 358 days for the wild-type group and 1,050.75 days for the mutated group, p=0.099; OS of 555.14 days for the wild-type group and 1,441 days for the mutated group, p=0.056).

For the ATRX mutation, there was no difference in PFS (median time of 715.4 days if preserved expression was observed and 382.96 days if there was loss of expression, p=0.3) or OS (median time 1,100.3 days if retained expression and 694.71 days if loss of expression, p=0.167).

There was no difference in PFS (296.26 days for samples with less than 10% p53-positive cells and 508.61 days for samples with more than 10% p53-positive cells) (p=0.17). OS was 584.71 days when less than 10% of cells were positive and 692.71 days when more than 10% of cells were positive (p=0.26).

PFS was 480.5 days in the group with a ki67 index less than 25%, and PFS was 371.05 days in the group with a ki67 index greater than 25%; moreover, there was no difference between the two groups (p=0.64). OS was 782.08 in the group with a ki67 index less than 25% and 683.37 in the group with a ki67 index greater than 25% (p=0.95).

MGMT status was analyzed in 25 samples. Moreover, there was no significant difference in prognosis, with a PFS of 421.14 days in the unmethylated group and 663.63 days in the overmethylated group (p=0.43). The OS was 626.92 days in the unmethylated group and 1,075.25 days in the overmethylated group (p=0.24).

**Figure 1:**
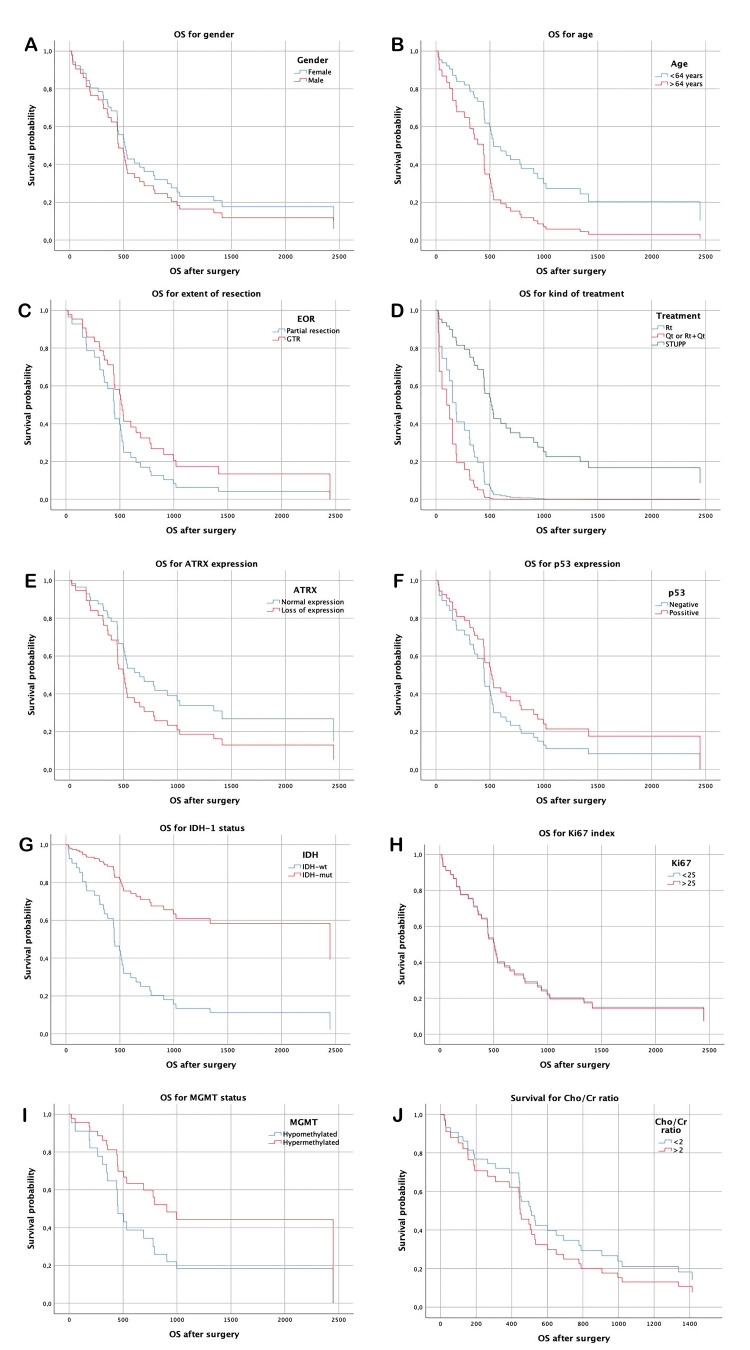
OS analysis according to clinical and histological characteristics (A) Overall survival, difference for gender, (B) age over 64 years, (C) extent of resection, (D) treatment group, (E) ATRX expression, (F) p53 expression, (G) IDH mutation, (H) Ki67 proliferation index, (I) MGMT mutation status, (J) Ratio Cho/Cr by spectroscopy. OS: overall survival (expressed in days from surgery to death), IDH-1: isocitrate dehydrogenase 1, MGMT: O-6-methylguanine-DNA-methyltransferase, Cho: choline, Cr: creatine

**Figure 2:**
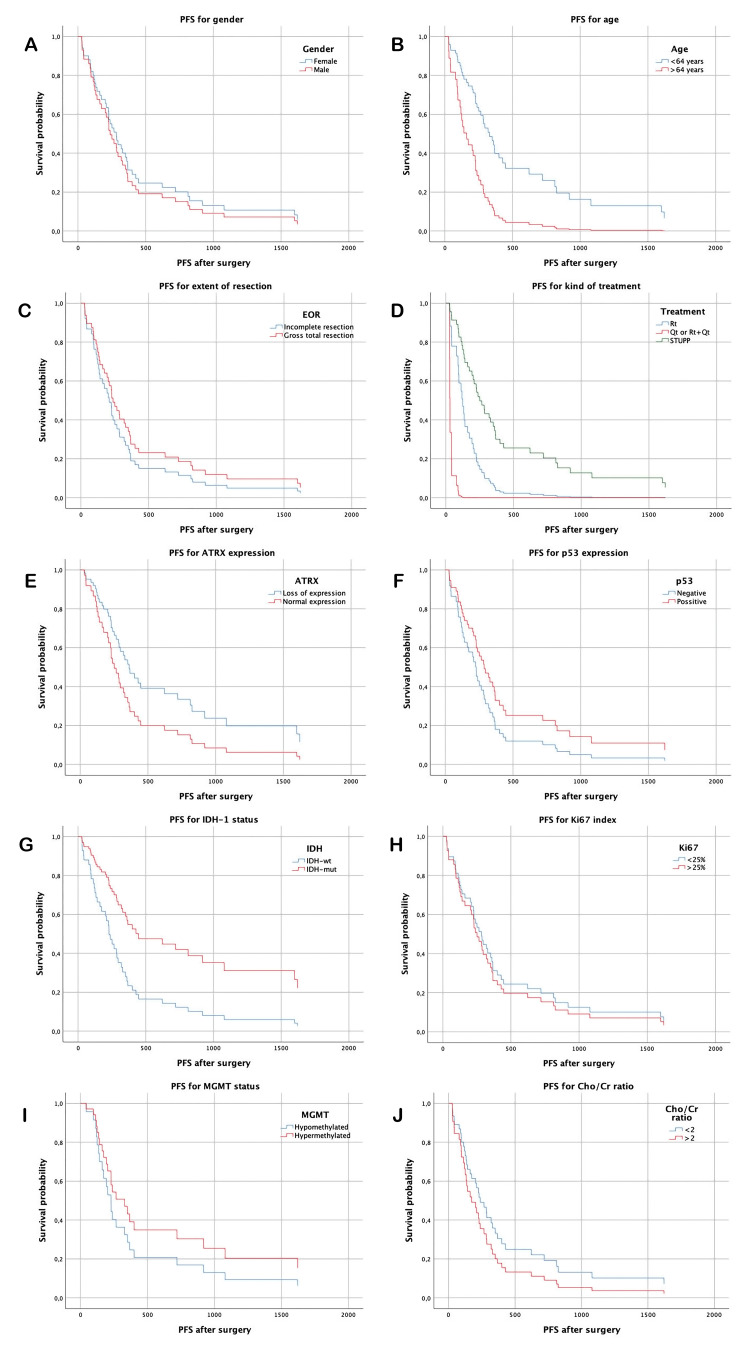
PFS analysis according to clinical and histological characteristics (A) Progression-free survival, difference for gender, (B) age over 64 years, (C) extent of resection, (D) treatment group, (E) ATRX expression, (F) p53 expression, (G) IDH mutation, (H) Ki67 proliferation index, (I) MGMT mutation status, (J) ratio Cho/Cr by spectroscopy PFS: progression-free survival (expressed in days from surgery to recurrence or disease progression), IDH-1: isocitrate dehydrogenase 1, MGMT: O-6-methylguanine-DNA-methyltransferase, Cho: choline, Cr: creatine

Spectroscopy was performed in 39 patients, with a Cho/Cr ratio greater than 2 in 15 patients. The prognosis was not different between the two groups, with a PFS of 444.83 days for which the ratio was <2 and 308.47 days for which the ratio was >2 (p=0.27). The mean OS was 677.42 days in patients with a BMI <2 and 530.76 days in patients with a BMI <2 (p=0.44) (Table 2, Figures 1A-1J, 2A-2J).

**Table 2:** Univariate analysis in terms of progression-free survival and overall survival of the main clinical characteristics taken into consideration HR: Hazard ratio, CI: Confidence interval, RT: radiotherapy, CT: chemotherapy, IDH-1: isocitrate dehydrogenase 1, MGMT: O-6-methylguanine-DNA-methyltransferase, Cho: choline, Cr: creatine

	n	PFS			OS		
		Mean (days)	HR (95% CI)	P-value	Mean (days)	HR (95% CI)	P-value
	46	428.61			734.02		
Sex							
Female	21	449.71	1		732.57	1	
Male	25	407.68	1.18 (0.65-2.14)	0.58	735.24	1.23 (0.65-2.32)	0.51
Age							
<64 years	30	554.90	1		889.9	1	
³64 years	16	191.81	2.76 (1.43-5.36)	0.003	441.75	2.19 (1.13-4.24)	0.019
Surgery							
Radical	37	440.10	1		793.32	1	
Partial/biopsy	9	372.44	1.29 (0.59-2.79)	0.51	490.22	1.73 (0.79-3.79)	0.19
Treatment							
RT or none	4	132.25	1		161.00	1	
RT-CT or CT alone	1	31	8.28 (0.64-106.37)	0.104	153.00	1.83 (0.18-18.17)	0.60
Stupp regimen	41	473.17	0.34 (0.11-0.97)	0.045	804.45	0.22 (0.07-0.67)	0.007
IDH-1							
Wild-type	42	358	1		555.14	1	
Mutated	4	1050.75	0.423 (0.14-1.17)	0.099	1441	0.24 (0.058-1.039)	0.056
MGMT methylation							
<29%	19	421.14	1		626.92	1	
>29%	6	663.63	0.67 (0.24-1.82)	0.43	1075.25	0.48 (0.13-1.66)	0.24
ATRX							
Normal expression	10	715.4	1		1100.3	1	
Loss of expression	31	382.96	1.71 (0.79-3.70)	0.167	694.71	1.55 (0.67-3.57)	0.30
p53							
<10% positive cells	23	296.26	1		584.71	1	
>10% possitive cells	21	508.61	0.64 (0.34-1.2)	0.17	692.71	0.69 (0.36-1.32)	0.26
Ki67							
< 25% positive cells	26	480.5	1		782.08	1	
>25% positive cells	19	371.05	1.15 (0.62-2.12)	0.64	683.37	1.02 (0.53-1.94)	0.95
Cho/Cr ratio							
<2	24	444.83	1		677.42	1	
>2	15	308.47	1.45 (0.74-2.83)	0.27	530.76	1.309 (0.65-2.6)	0.44

**Figure 3:**
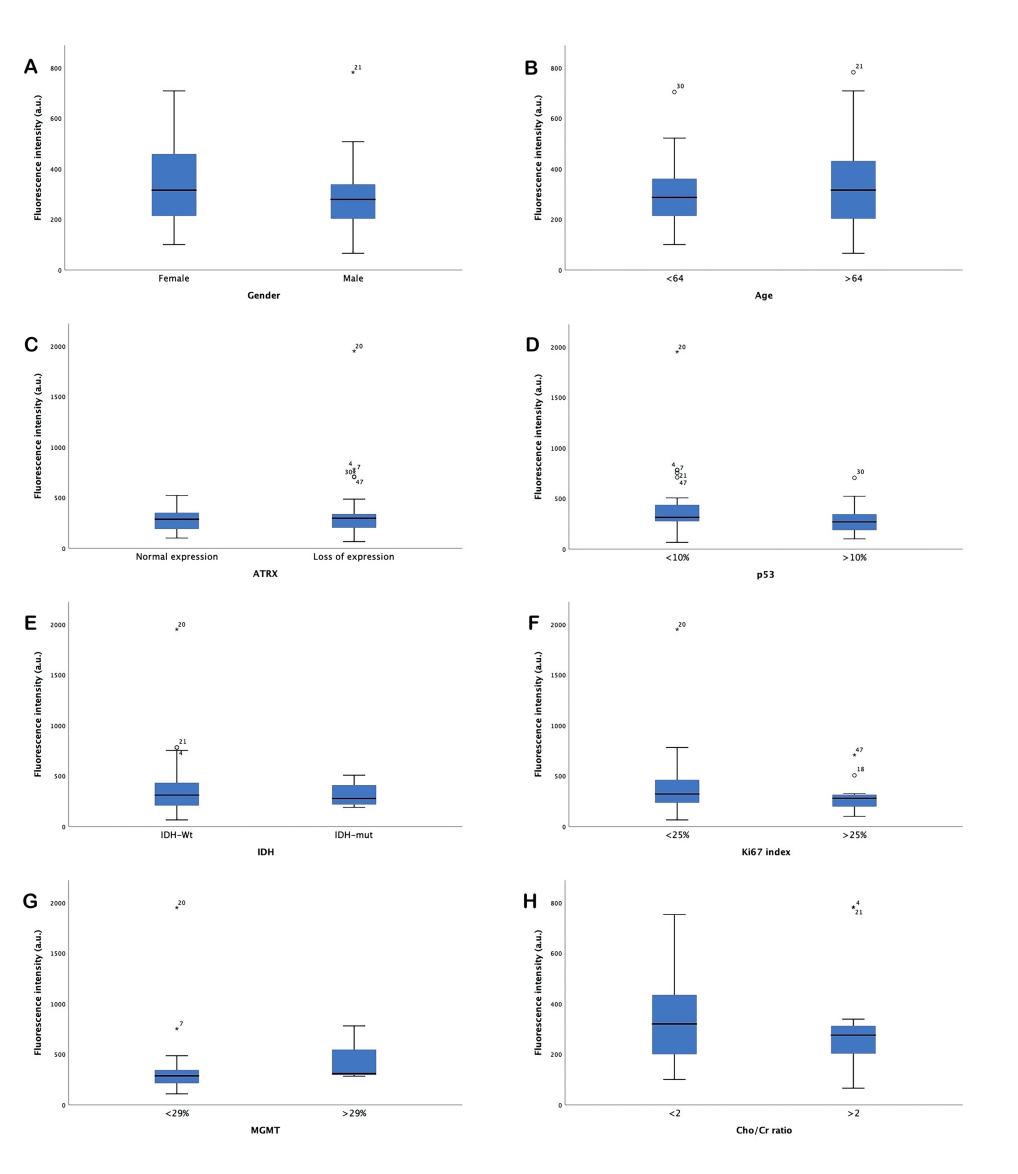
Mean fluorescence for patient and histological characteristics (A) Mean fluorescence for gender, (B) for group of age, (C) ATRX expression, (D) p53 expression, (E) IDH mutation, (F) Ki67 proliferation index, (G) MGMT mutation status, (H) Ratio Cho/Cr by spectroscopy. IDH: isocitrate dehydrogenase, MGMT: 6-O-methylguanine-DNA-methyltansferase, Cho: coline, Cr: creatine. Fluorescence intensity is measured in arbitrary units (a.u.)

The mean fluorescence intensity of the samples was 367.6 a.u., with a minimum of 65.84 a.u. and a maximum of 1,949.7 a.u., with no difference in the intensity according to sex (mean fluorescence for females of 352.18 a.u., 298.76 a.u. for males, p=0.35). There was no difference in age (311.18 a.u. in the >64 years group, 339.41 a.u. in the >64 years group; p=0.63). For histological characteristics, in Ki67 index expression <25% the mean intensity was 411.69 a.u. and in >25% was 283.57 a.u., with no significant difference (p=0.17). The mean fluorescence intensity was 294.94 a.u. for the group with normal ATRX expression, compared to 368.62 a.u. for the group with loss of expression (p=0.52). For p53 expression, there was no difference in fluorescence intensity (421.67 a.u. in low expression and 297.33 a.u. in high expression, p=0.6). IDH mutation status did not significantly differ in fluorescence intensity (372.68 a.u. in the wild-type group and 313.12 a.u. in the mutated group, p=0.7). The MGMT methylation status was not related to fluorescence intensity (264.74 a.u. in the unmethylated group and 420.61 a.u. in the hypermethylated group; p=0.28). The Cho/Cr ratio was not related to fluorescence intensity (342.08 a.u. if the ratio was <2 and 316.08 a.u. if the ratio was >2, p=0.67) (Table 3, Figures 3A-3H).

**Table 3:** Mean fluorescence for clinical and histopathological characteristics IDH-1: isocitrate dehydrogenase 1, MGMT: O-6-methylguanine-DNA-methyltransferase, Cho: coline, Cr: creatine. Fluorescence intensity is expressed in arbitrary units (a.u.)

	N	Mean fluorescence (a.u.)	SD	P-value
Gender				
Female	16	352.18	180.65	0.35
Male	21	298.76	162.49	
Age				
<64 years	23	311.18	145.58	0.63
>64 years	14	339.41	209.45	
Ki67				
<25%	31	411.69	344.68	0.17
>25%	15	283.57	153.35	
ATRX				
Normal expression	9	294.94	152.47	0.52
Loss of expression	34	368.62	332.24	
p53				
<10% positive cells	27	421.67	361.93	0.16
>10% possitive cells	19	297.33	162.74	
IDH				
IDH-wt	43	372.68	308.60	0.70
IDH-mut	4	313.12	138.85	
MGMT				
<29%	15	264.74	94.61	0.28
>29%	4	420.61	240.35	
Cho/Cr ratio				
<2	28	342.08	175.21	0.67
>2	14	316.08	211.48	

**Figure 4:**
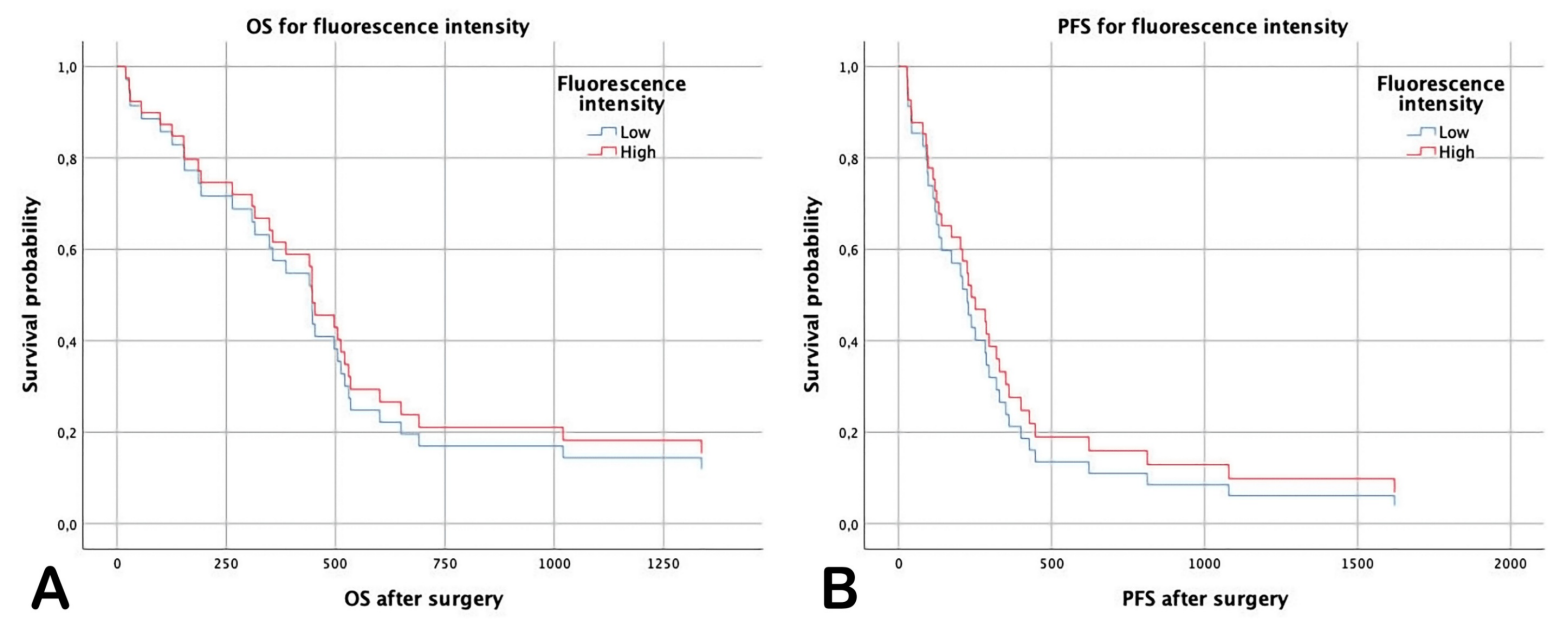
Analysis of survival according to fluorescence intensity (A) Overall survival for fluorescence intensity, (B) progression-free survival for fluorescence intensity. Indicates the days from surgery until death and recurrence or progression. OS: overall survival, PFS: progression-free survival

Survival analysis for PFS revealed no difference between the groups with high and low fluorescence intensities (296.68 vs 451.72 days, HR=0.74, 95% CI=0.37-1.47, p=0.39). The mean OS times of patients in the low-intensity fluorescence group were 539.11 days and 613.06 days, respectively, while those in the high-intensity fluorescence group were 0.87 (95% CI=0.43-1.76, p=0.71) (Table 4, Figures 4A, 4B).

**Table 4:** Survival analysis for fluorescence HR: hazard ratio, CI: confidence interval

	n	PFS			OS		
		Mean (days)	HR (95% CI)	P-value	Mean (days)	HR (95% CI)	P-value
Fluorescence intensity							
Low	19	296.68	1		539.11	1	
High	18	451.72	0.747 (0.37-1.47)	0.39	613.06	0.879 (0.43-1.76)	0.71

**Figure 5:**
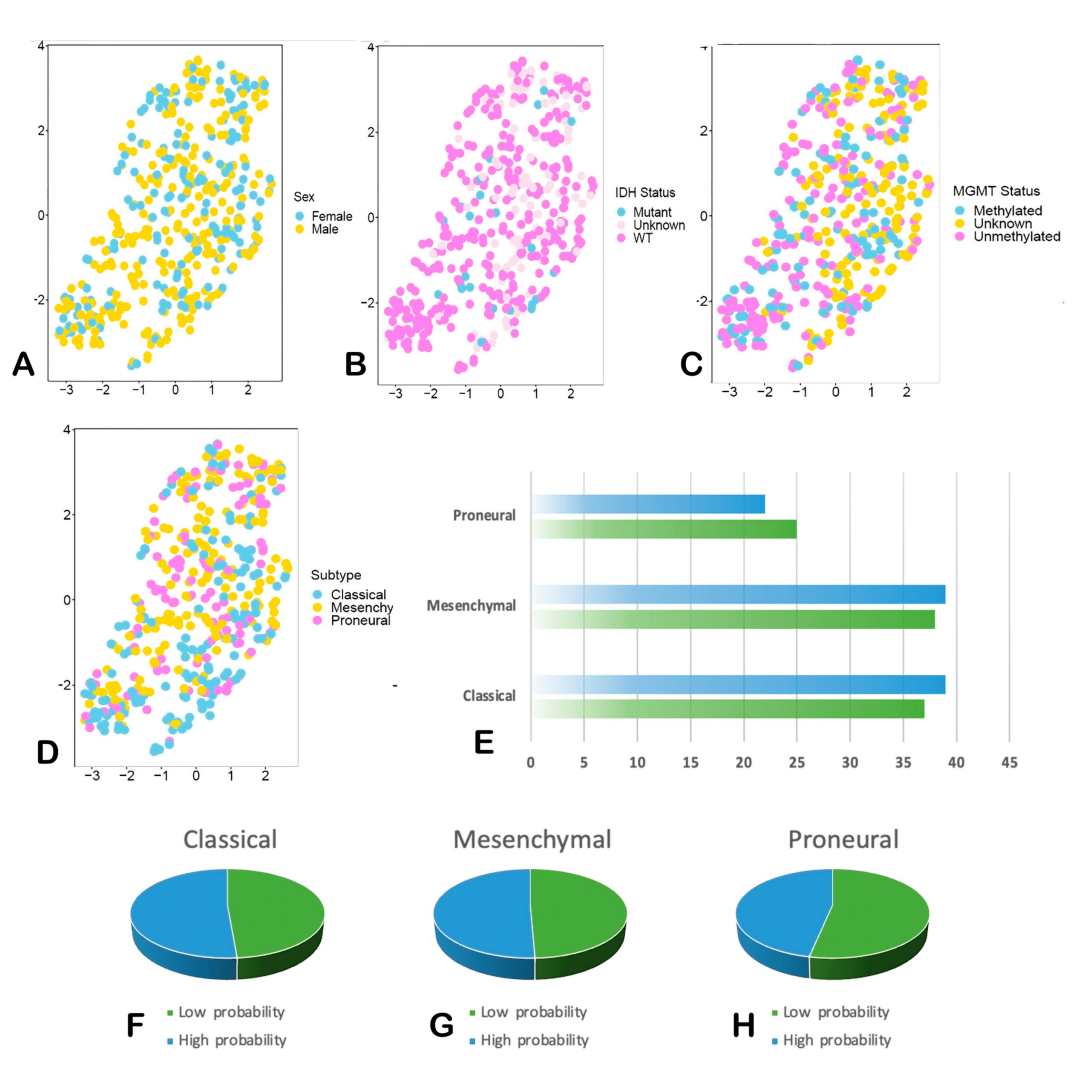
Synthetic centroid represents the likelihood to intake 5-ALA Uniform Manifold Approximation and Projection (UMAP) dimension reduction analysis. (A) UMAP analysis of fluorescence probability according to gender, (B) IDH-1 mutation status, (C) MGMT methylation, (D) molecular subtype of HGG. (E) patients with higher and lower probability of fluorescence according to protein expression involved in 5-ALA metabolism for molecular subtype, for the (F) proneural, (G) mesenchymal and (H) classical subtype. IDH-1: Isocitrate dehydrogenase, MGMT O-6-methylguanil-DNA-methyltransferase, HGG: high grade glioma, 5-ALA: 5-aminolevulinic acid

**Figure 6:**
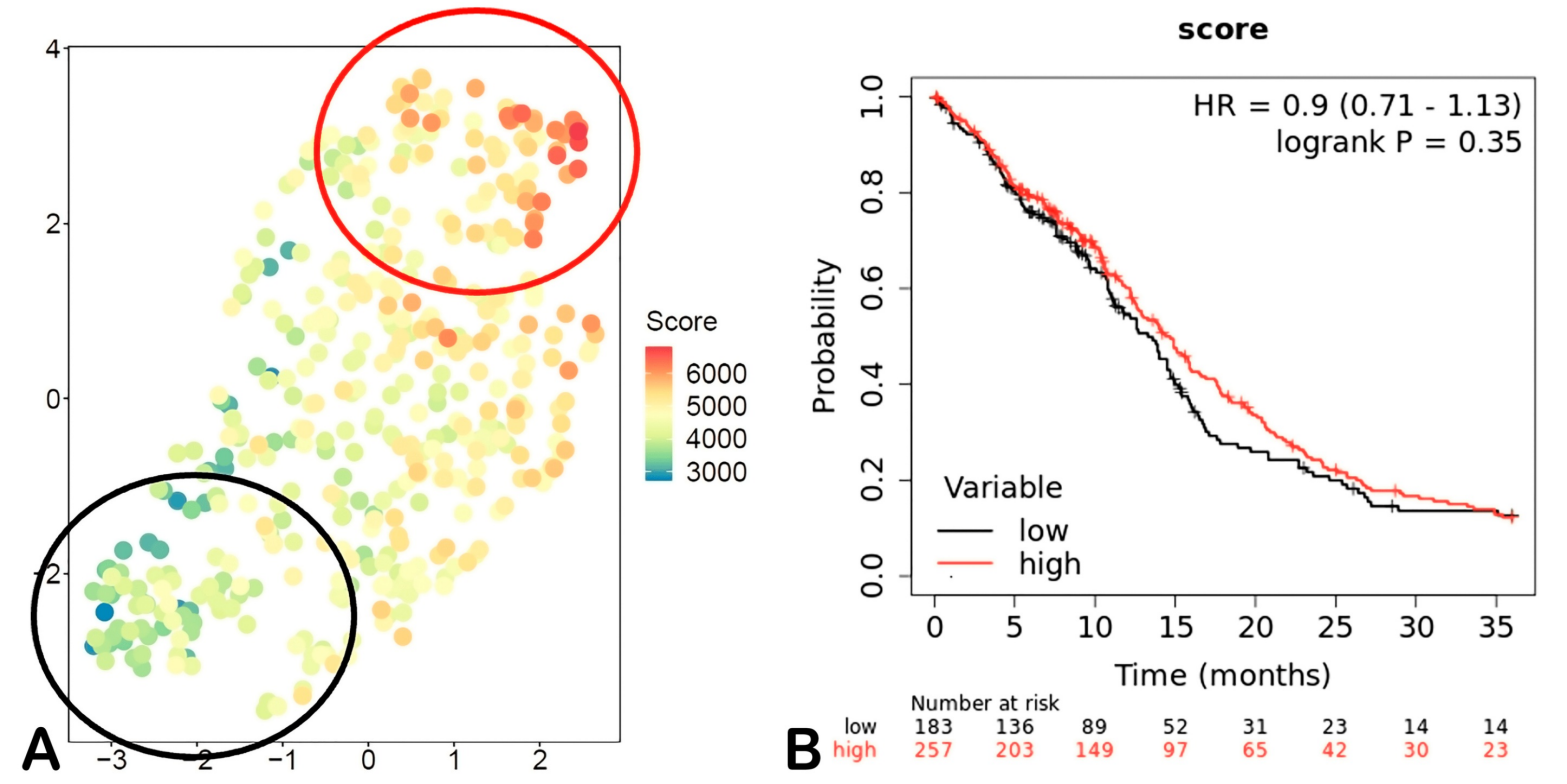
Synthetic centroid sample for survival probability according to UMAP analysis (A) Selection of higher fluorescence probability population (red circle) and lower fluorescence probability (black circle). (B) Survival analysis of higher and lower fluorescence probability. UMAP: uniform manifold approximation and projection, HR: hazard ratio

UMAP analysis of the expression of genes affecting fluorescence intensity using HGG ATAC-seq data from The Cancer Genome Atlas revealed that the proneural molecular subtype had a lower probability of exhibiting high fluorescence intensity, while the classical and mesenchymal subtypes had a slightly greater probability of exhibiting high fluorescence intensity. Survival analysis of the groups with a high or low probability of high fluorescence intensity according to gene expression showed no difference (HR 0.9, 95% CI 0.71-1.13; p=0.35) (Figures 5A-5H, 6A, 6B).

## Discussion

Previous studies have shown an association between histological tumor characteristics and prognosis [12], with IDH mutation being the strongest independent predictor; however, many other genomic alterations and gene expression have been analyzed, and combinations of different mutations have been studied [13]. ATRX is involved in genomic stability and cell division regulation. Loss of ATRX expression has been associated with improved outcomes [13], although no difference was found among the other studies [14]. In our cohort, there was no association between survival and ATRX status.

In primary HGG, p53 mutation is observed during disease progression after treatment. The relationship of progression to disease has been controversial; for example, some authors associate it with a good prognosis [15,16], but others have found that p53 mutation is related to poor prognosis, especially in older people [13].

The Ki67 labeling index is a cell proliferation marker and has been associated with tumor grade in glioma. A greater percentage of HGG-positive cells is observed than in diffuse astrocytoma [17]. Several studies have shown that a higher proliferation index is associated with poor prognosis [18,19], but other studies have failed to show this difference [19-21]. Dahlrot et al. analyzed the effect of eliminating Ki67-positive cells on survival by observing replicating glia, and they found no relation to survival [20]. In our sample, there was no relationship with survival.

IDH mutation is the most important factor associated with prognosis and is a hallmark of the classification criteria in the current WHO grading system. IDH mutation is associated with longer survival independent of other factors, such as MGMT methylation status, vascular proliferation, or necrosis [22]. According to our results, the number of patients with IDH1 mutations was low, so there was no significant difference in OS or PFS.

MGMT promoter hypermethylation increases tumor responsiveness to treatment with temozolomide. Moreover, MGMT methylation does not strongly correlate with MGMT protein expression, suggesting that other epigenetic factors are associated with MGMT expression [23]. Studies of survival associated with MGMT methylation have produced different results [24,25], and in our study, there was no difference between patients with >29% methylation and those with less methylation. The lack of relation between the mutations and prognosis in this study can be explained by the sample size and the selection bias, as the patients included in the study were the ones in a good clinical status to be operated and receive complementary treatment, leaving patients with worse clinical status out of the study.

The fluorescence heterogeneity produced by 5-ALA is still unclear. Many factors related to protoporphyrin metabolism have been proposed to influence fluorescence variability [26]. We searched for differences in fluorescence intensity according to common mutations routinely analyzed by immunochemistry in HGG samples. To avoid differences in fluorescence intensity samples were obtained after four to five hours of 5-ALA intake and processed in the same conditions. We found no difference in fluorescence intensity according to sex or age. By analyzing IDH-1 status, Kim et al. demonstrated increased fluorescence intensity in WHO III gliomas with IDH-1 mutations [4]; in contrast, Ohba et al. found no difference in fluorescence positivity in WHO grade IV gliomas according to IDH-1 status, but no quantification of the fluorescence was performed [27]. In our cohort, there was no difference in fluorescence intensity according to IDH-1 status.

We analyzed the fluorescence intensity according to the proliferation index and found no difference between the groups. Previous studies have shown positive fluorescence in WHO Grade II and III gliomas with a high proliferation index [3,28]. We found no association between fluorescence intensity and ATRX, p53, or MGMT methylation status, and no previous studies have compared these results.

Currently, no study has quantified the fluorescence intensity in HGG as a predictor of survival. We found no difference in PFS or OS according to fluorescence intensity, which was also the case for UMAP analysis of the expression of genes affecting fluorescence intensity using TCGA data. According to the analysis of gene expression, greater fluorescence intensity was likely observed in the mesenchymal and classic HGG subtypes. Almiron et al. analyzed gene expression in fluorescent and nonfluorescent samples and found no difference in the expression of genes related to Haem metabolism but did detect high expression of genes related to growth, survival, and angiogenesis in fluorescent tumors. The Cancer Genome Atlas data for HGG revealed that, in tumors with higher fluorescence scores, the presence of mutations in ABCC9 was related to 5-ALA efflux, and a low fluorescence score was associated with mutations in ATRX and IDH. For the genetic profile, they found the neural subtype to have a lower fluorescence score, as in our analysis [29]. In contrast to their fluorescence score, we did not observe differences in fluorescence between patients with ATRX or IDH mutations.

Our study is the first to quantify the fluorescence of tumor samples and compare it to histological characteristics. Previous studies have used qualitative criteria, such as high, medium, or low fluorescence according to intraoperative findings. HGGs exhibit wide variability in gene expression and mutation, and fluorescence is affected not only by protein expression but also by metabolic tumor status, which varies during progression; moreover, fluorescence analysis cannot be performed without controlling for all the variables. In this way, a study using cell cultures, where the metabolic status of the tumor can be better controlled, can provide more information about the effects that molecular tumor profile can have on fluorescence intensity. With our results, we think that it is possible that the intensity of fluorescence does not predict a specific HGG subtype and that this is not related to patient prognosis.

Limitations

Survival analysis is limited to patients who are in a clinical situation suitable for surgery, which could affect the results. The analysis of the genes associated with fluorescence was performed in the Cancer Genome Atlas, and there was no fluorescence analysis in this database. The analysis of mRNA expression is a better procedure for identifying a correct relationship, and this could be the next step for this study.

## Conclusions

The fluorescence intensity in HGG varies within a single tumor, which reflects the high variability in gene expression and mutation. Higher fluorescence intensity is not related to a worse prognosis, as many factors are involved in the variability of fluorescence intensity that affects tumor metabolism. Identifying the principal factor affecting fluorescence in this tumor will help to improve tumor visualization during surgical resection, and this technique could also be used as a tool for phototherapy. A greater number of patients for sample analysis can provide a better understanding of fluorescence behavior, and quantification of fluorescence intensity avoids subjective bias, which will provide a more precise result. Cell culture can aid in understanding the influence of gene expression on PPIX accumulation, but a complete analysis of cellular metabolism is needed for accurate prediction of fluorescence; this process is not precise in culture settings.
